# RAGE-dependent mitochondria pathway: a novel target of silibinin against apoptosis of osteoblastic cells induced by advanced glycation end products

**DOI:** 10.1038/s41419-018-0718-3

**Published:** 2018-06-04

**Authors:** Y. X. Mao, W. J. Cai, X. Y. Sun, P. P. Dai, X. M. Li, Q. Wang, X. L. Huang, B. He, P. P. Wang, G. Wu, J. F. Ma, S. B. Huang

**Affiliations:** 1 Department of Prosthodontics, School and Hospital of Stomatology, Wenzhou Medical University, Wenzhou, People’s Republic of China; 2 Institute of Stomatology, School and Hospital of Stomatology, Wenzhou Medical University, Wenzhou, People’s Republic of China; 3 Department of Oral Implantology and Prosthetic Dentistry, Academic Centre for Dentistry Amsterdam (ACTA), MOVE Research Institute, University of Amsterdam and Vrije University Amsterdam, Amsterdam, 1081 LA The Netherlands; 4 Department of Stomatology, Taizhou Hospital, Wenzhou Medical University, Linhai, People’s Republic of China; 5 Division of General Dentistry, Eastman Institute for Oral Health, University of Rochester, Rochester, NY USA; 6 Protein Section, Laboratory of Metabolism, Center for Cancer Research, National Cancer Institute, National Institutes of Health, Bethesda, MA USA; 7 Department of Periodontology, Guanghua School of Stomatology, Sun Yat-sen University, Guangzhou, People’s Republic of China

## Abstract

Advanced glycation end products (AGEs) can stimulate osteoblast apoptosis and have a critical role in the pathophysiology of diabetic osteoporosis. Mitochondrial abnormalities are closely related to osteoblast dysfunction. However, it remains unclear whether mitochondrial abnormalities are involved in AGE-induced osteoblastic cell apoptosis. Silibinin, a major flavonolignan compound of silimarin, has strong antioxidant and mitochondria-protective properties. In the present study, we explored the possible mitochondrial mechanisms underlying AGE-induced apoptosis of osteoblastic cells and the effect of silibinin on osteoblastic cell apoptosis. We demonstrated that mitochondrial abnormalities largely contributed to AGE-induced apoptosis of osteoblastic cells, as evidenced by enhanced mitochondrial oxidative stress, conspicuous reduction in mitochondrial membrane potential and adenosine triphosphate production, abnormal mitochondrial morphology, and altered mitochondrial dynamics. These AGE-induced mitochondrial abnormalities were mainly mediated by the receptor of AGEs (RAGE). In addition, we found that silibinin directly downregulated the expression of RAGE and modulated RAGE-mediated mitochondrial pathways, thereby preventing AGE-induced apoptosis of osteoblastic cells. This study not only provides a new insight into the mitochondrial mechanisms underlying AGE-induced osteoblastic cell apoptosis, but also lays a foundation for the clinical use of silibinin for the prevention or treatment of diabetic osteoporosis.

## Introduction

Diabetes mellitus is a highly prevalent disease characterized by sustained hyperglycemia. It is closely associated with various complications, one of which is bone disease, such as osteoporosis^1^. Osteoporosis is a systemic skeletal disorder characterized by decreased mass and architectural deterioration of bone tissues^2^. Studies have reported greater risk of osteoporotic bone fractures in diabetic patients compared with the general population^1^. Given the prevalence of diabetic osteoporosis, there is an urgent need for better understanding of the molecular mechanisms underlying this pathological condition.

Recent research has suggested that advanced glycation end products (AGEs), senescent macroprotein derivatives formed at an accelerated rate in diabetes, participate in the pathological processes of various diabetic complications^3,4^, including diabetic osteoporosis^5^ and osteopenia^6^. Osteoblast apoptosis has a crucial role in bone development and maintenance^7^, and inhibition of diabetes-enhanced osteoblast apoptosis significantly improves new bone formation^8^. AGEs can induce osteoblast apoptosis. The AGEs-induced apoptosis is found to be highly related to interaction with its main receptor of AGEs (RAGE). Many signaling pathways, such as MAPK cascade, participate in this process^9,10^. However, the mechanisms linking RAGE activation to osteoblast apoptosis are still not completely understood. In cells such as adipocytes and retinal pigmented epithelium cells, the activation of the AGE-RAGE axis enhances oxidative stress (OS), affects mitochondrial function, and ultimately influences cell metabolism under various pathological conditions^11,12^. OS is characterized by the overproduction of reactive oxygen species (ROS). Mitochondria are a major source of ROS and also the principal target of ROS attack^13^. Mitochondrial dysfunction influences osteoblast function^14^ and has been identified as a key mechanism leading to OS-induced apoptosis of osteoblastic cells^15^. Whether AGE-RAGE-related OS and mitochondrial abnormalities are involved in the AGE-induced apoptosis of osteoblastic cells needs further exploration.

Mitochondria are dynamic organelles that undergo continuous fission and fusion. Fission are regulated by dynamin-related protein 1 (Drp1) and fission 1 (Fis1), while fusion are regulated by large dynamin-related GTPases known as mitofusins (Mfn1 and Mfn2) as well as optic atrophy 1 (Opa1)^16^. Our previous findings indicated that mitochondrial dynamic alterations significantly affected mitochondrial function, number, and shape under diabetic conditions^17^. Furthermore, impaired mitochondrial dynamics contribute substantially to OS-induced osteoblast injury^18^ and cell apoptosis^19^. A few studies have indicated that the AGE-RAGE axis mediates mitochondrial dysfunction and altered mitochondrial dynamics in pancreaticβ-cells^20^ and high-fat fed mice^21^. On the basis of these findings, we hypothesized that mitochondrial OS, dysfunction, and altered dynamics could be critical reasons for AGE-induced osteoblastic cell apoptosis.

Silibinin, a major flavonolignan compound of silimarin, demonstrates strong antioxidant properties and effectively prevents oxidative damage in various diabetic complications^22,23^. Silibinin also protects mitochondria by restoring mitochondrial potential, respiration, and membrane integrity^24–26^. Furthermore, silibinin exerts bone-forming and osteoprotective effects, and attenuates bone loss in diabetes-related bone diseases^27–29^. Despite the broad spectrum of pharmacological activities of silibinin, whether silibinin can afford protection against AGE-induced apoptosis of osteoblastic cells, and the possible underlying mechanisms of such an effect, remain to be investigated.

The aims of the present study were to investigate (1) whether mitochondrial OS, dysfunction, and dynamic alterations are involved in AGE-induced apoptosis of osteoblastic cells; (2) the pathological role of RAGE in AGE-induced osteoblastic cell apoptosis and related mitochondrial molecular pathways; (3) the cytoprotective potential of silibinin against AGE-elicited apoptosis of osteoblastic cells; and (4) the mechanism underlying the protective effects of silibinin. For the first time, we demonstrated that RAGE-dependent mitochondrial abnormalities contributed to AGE-induced apoptosis of osteoblastic cells. Furthermore, silibinin directly downregulated the RAGE expression, attenuated RAGE-mediated mitochondrial damage, thereby preventing AGE-induced apoptosis of osteoblastic cells. This study provides a new insight into the mitochondrial mechanisms underlying AGE-induced osteoblastic cell apoptosis and the protective effect of silibinin on diabetic osteoporosis.

## Results

### AGEs enhanced apoptosis of osteoblastic MC3T3-E1 cells

**Fig. 1:**
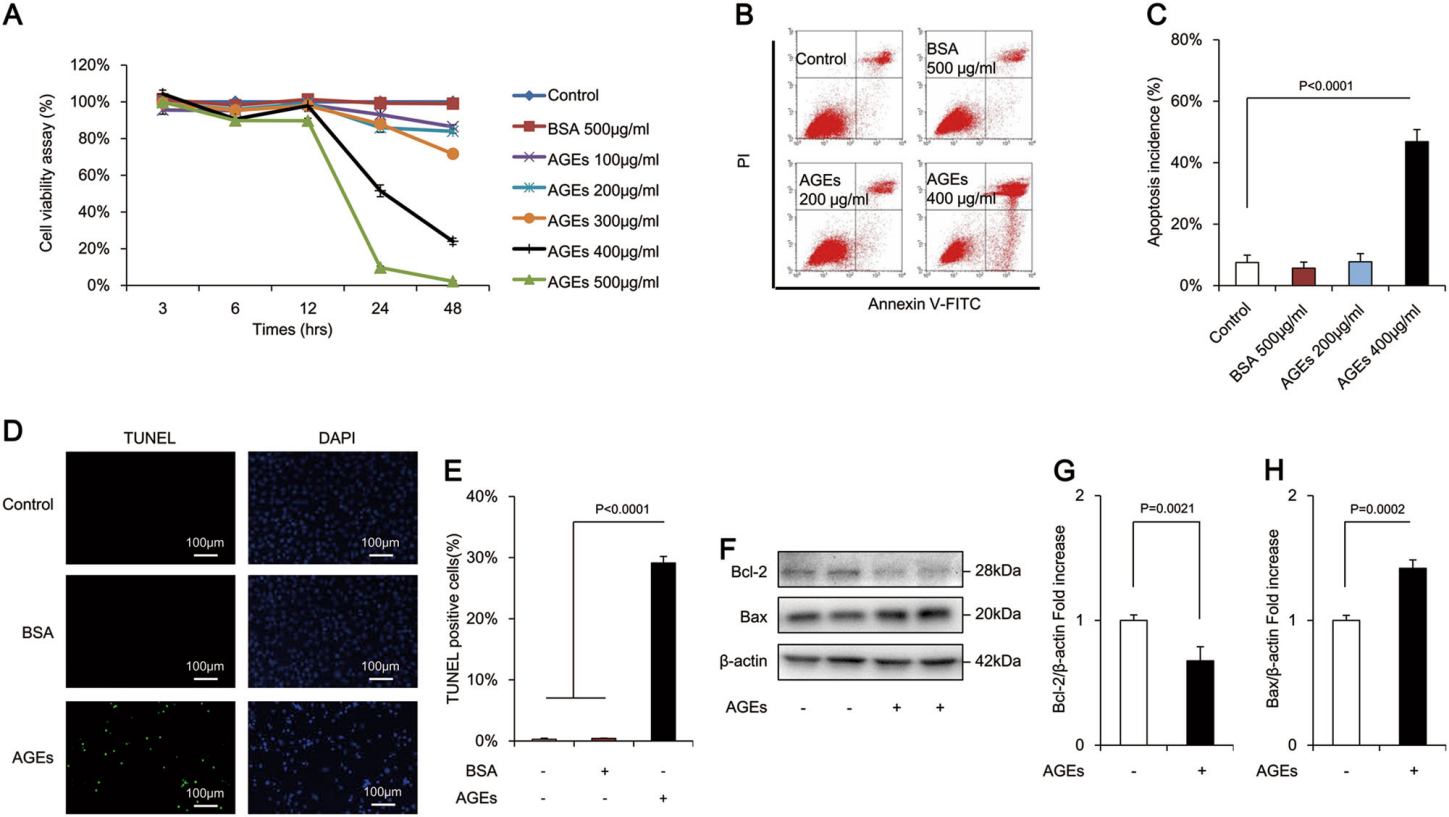
AGEs-induced apoptosis in osteoblastic MC3T3-E1 cells. **a** Cell viability was determined by MTT reduction in osteoblastic cells in the presence of AGEs. Error bars indicate SEM (*n* = 6). **b**, **c** Flow cytometric quantification of apoptosis. Error bars indicate SEM (*n* = 6). **d**, **e** TUNEL staining and assay. Error bars indicate SEM (*n* = 300). Scale bars = 100 μm. **f** Representative immunoreactive bands for Bcl-2 and Bax in osteoblastic cells in the presence of AGEs. Full-length blots are presented in Supplementary Figure 4. **g** Quantification of immunoreactive bands for Bcl-2 and Bax relative to β-actin. Error bars indicate SEM (*n* = 6)

AGEs in the concentration range of 100–500 μg/mL significantly decreased the cell viability in a time- and dose-dependent manner (Fig. 1a). The cell viability of osteoblastic MC3T3-E1 cells treated with 500 μg/mL AGEs was nearly eliminated. Thus, an AGE concentration of 400 μg/mL (for 24 h) was used for subsequent experiments. To further confirm the effects of AGEs on osteoblastic MC3T3-E1 cells, the production of LDH, a biomarker of a measurement of cytotoxicity, were also examined. The results showed that there was a significant increase in LDH release following exposure of the cells to 400 μg/mL AGEs (Supplementary Figure 1). Annexin/propidium iodide staining assay (an indicator of alterations in cell membrane permeability) revealed a dose-dependent increase in the incidence of apoptosis. Overall, 400 μg/mL AGEs significantly enhanced the rate of late apoptosis (defined as the last step of mitotic catastrophe) in comparison with the control group (Fig. 1b, c). The pro-apoptotic effects of AGEs were further verified by terminal deoxynucleotidyl transferase dUTP nick-end labeling (TUNEL) staining (an indicator of DNA damage) (Fig. 1d). The percentage of TUNEL-positive cells significantly increased by 100-fold after treatment with 400 μg/mL AGEs (Fig. 1e). Compared with the control group, AGEs dramatically decreased the level of anti-apoptotic Bcl-2 but increased pro-apoptotic Bax in MC3T3-E1 cells (Fig. 1f, g). These results indicated that the AGE-induced MC3T3-E1 cell death occurred primarily through apoptosis.

### AGE treatment resulted in mitochondrial OS, abnormal morphology, altered dynamics, and dysfunction in osteoblastic MC3T3-E1 cells

**Fig. 2:**
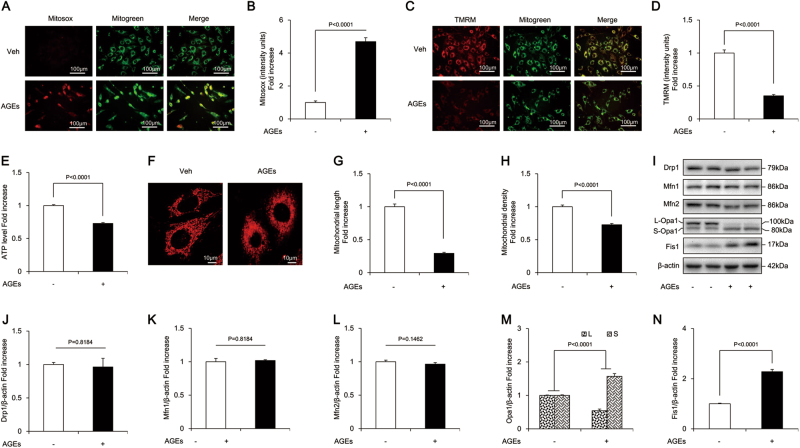
AGEs-induced mitochondrial abnormalities in osteoblastic MC3T3-E1 cells. **a**, **b** Representative images showing MitoSOX staining and quantification in the indicated groups. Scale bars = 100 μm. **c**, **d** Representative images with TMRM staining and quantification in the indicated groups. Scale bars = 100 μm. **e** ATP production in the indicated groups. **f**–**h** Representative images of mitochondrial morphology, and measurements of mitochondrial length and density in the indicated groups. Error bars indicate SEM (*n* = 15) Scale bars = 10 μm. **i**–**n** Representative immunoreactive bands and quantification of immunoreactive bands for Drp1, Mfn1, Mfn2, Opa1, and Fis1 in osteoblasts in the presence of AGES. Error bars indicate SEM (*n* = 6). Full-length blots are presented in Supplementary Figure 4

AGEs significantly increased the production of mitochondrial ROS (mtROS) (indicated by MitoSOX staining) (Fig. 2a, b) and markedly decreased mitochondrial membrane potential (indicated by tetramethylrhodaminemethylester [TMRM] staining) (Fig. 2c, d). The results were similar with the mitochondrial events resulted from administration of H_2_O_2_, which is widely used to establish the oxidative injury model (Supplementary Figure 2), suggesting the involvement of mitochondrial oxidative damage. In addition, AGEs caused severe mitochondrial dysfunction, as evidenced by reduced ATP (Fig. 2e). Furthermore, unlike from the regularly distributed, rod-like or elongated mitochondria in the control group, the mitochondria in the presence of AGEs were fragmented, misshapen, and bleb-like (Fig. 2f). The density and length of mitochondria (major axes) were significantly reduced by AGEs (Fig. 2g, h).

Furthermore, compared with the control cells, osteoblasts exposed to AGEs showed significantly increased expression of Fis1 (Fig. 2i, n), while no significant changes were found in Drp1, Mfn1, or Mfn2 (Fig. 2i–l). There are five splice variants of Opa1, which fall between 80 and 100 kDa in mass and are seen on western blotting as two doublets. AGEs reduced the level of the long form of Opa1 (L-Opa1) and increased the expression of its short form (S-Opa1) (Fig. 2i, m). These results suggested exacerbated mitochondrial fission in AGE-treated MC3T3-E1 cells, which might impair mitochondrial morphology and function, and subsequently lead to apoptosis.

### Effects of MitoQ, CsA, and silibinin on AGE-induced apoptosis of osteoblastic MC3T3-E1 cells

**Fig. 3:**
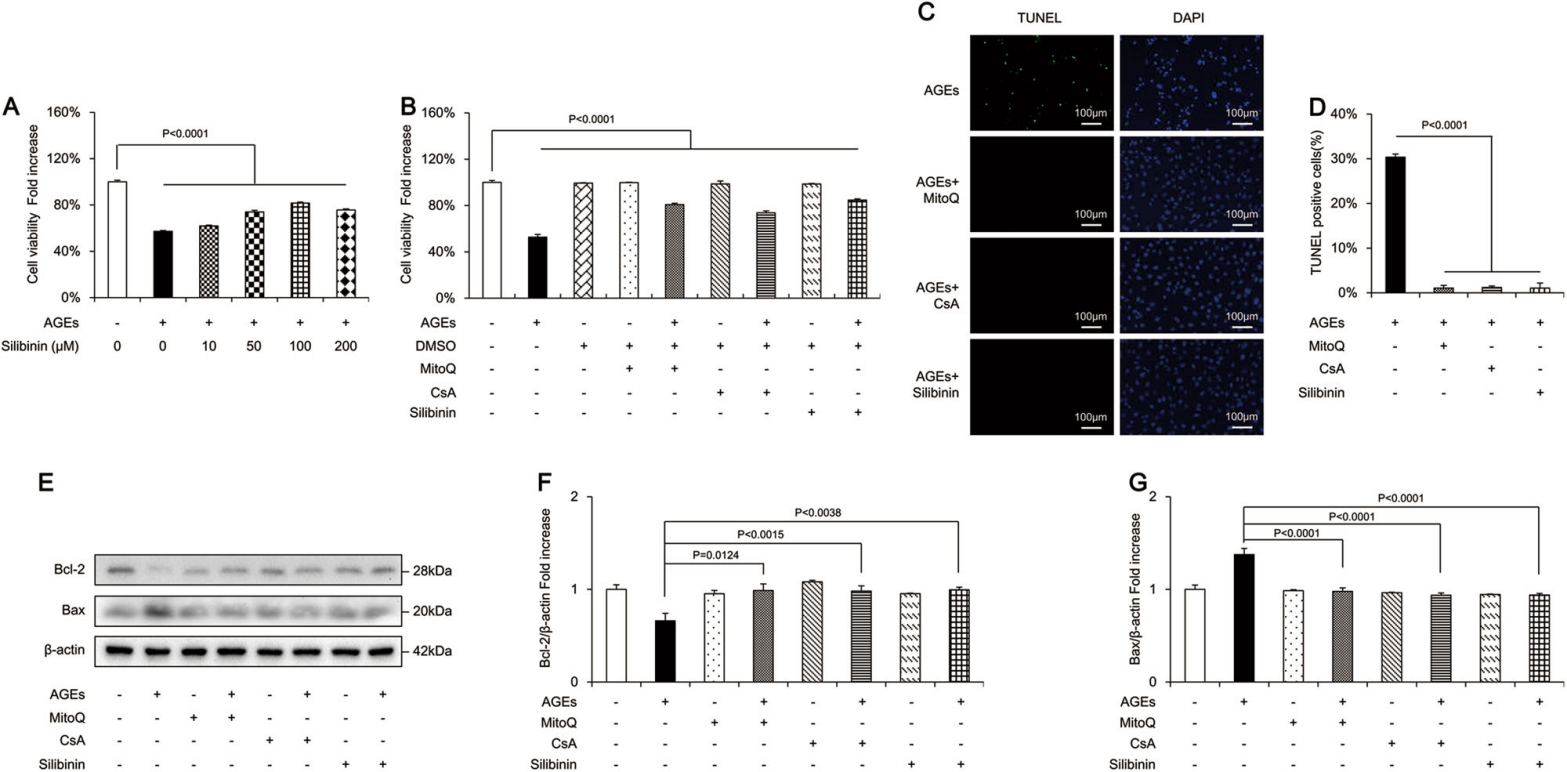
MitoQ, CsA, and silibinin attenuated AGEs-induced apoptosis in osteoblastic MC3T3-E1 cells. **a** Cell viability determined by MTT reduction in osteoblastic cells in the presence of AGEs with or without MitoQ or CsA. Error bars indicate SEM (*n* = 6). **b**, **c** TUNEL staining and assay after treatment with MitoQ or CsA. Error bars indicate SEM (*n* = 300). Scale bars = 100 μm. **d** Representative immunoreactive bands for Bcl-2 and Bax in osteoblastic cells with (+) or without (−) treatment of MitoQ or CsA in the presence of AGEs (+) or culture medium (−). **e**–**g** Quantification of immunoreactive bands for Bcl-2 and Bax relative to β-actin. Error bars indicate SEM (*n* = 6). Full-length blots are presented in Supplementary Figure 4

To further confirm the role of mitochondrial OS and dysfunction in AGE-induced osteoblastic cell apoptosis, we pretreated cells with MitoQ, a mitochondria-targeted antioxidant, along with AGEs. As shown in Fig. 3b and d, MitoQ significantly increased cell viability and attenuated cell apoptosis induced by AGEs. Furthermore, MitoQ antagonized the effects of AGEs by reversing the expression of Bcl-2 and Bax (Fig. 3e–g). These results indicated that mitochondrial OS was involved in the process of AGE-induced apoptosis of osteoblastic cells.

Loss of mitochondrial membrane potential has been reported as a common early marker and an essential event in apoptosis. Our results showed that mitochondrial membrane potential was significantly decreased in AGE-induced apoptosis of osteoblastic cells (Fig. 2c, d). Reduced mitochondrial membrane potential favors the formation and opening of the mitochondrial permeability transition pore (mPTP) through which mitochondrial apoptotic proteins are released into the cytosol and mediate apoptosis of various cell types^30–32^. To explore the role of mPTP opening in AGE-induced apoptosis of osteoblastic cells, we applied a well-established pharmacological mPTP blocker, CsA. We found that CsA significantly increased cell viability and reduced the cell apoptosis induced by AGEs (Fig. 3b–d). CsA also upregulated Bcl-2 protein expression and downregulated the Bax level (Fig. 3e–g). These results revealed that the loss of mitochondrial membrane potential involved in AGE-induced apoptosis in osteoblastic MC3T3-E1 cells was dependent on mPTP opening.

We further investigated whether silibinin could afford protection against AGE-induced osteoblast apoptosis. We treated MC3T3-E1 cells with various concentrations of silibinin prior to AGE treatment. As shown in Fig. 3a and b, silibinin (100 μM, 24 h) was not cytotoxic and significantly increased the viability of MC3T3-E1 cells in a dose-dependent manner. However, no obvious difference was observed between silibinin concentrations of 100 and 200 μM. Thus, we chose 100 μM as our final experimental concentration. Silibinin also significantly decreased the LDH release induced by AGEs, affirmed that silibinin had a protective effect on MC3T3-E1 cells (Supplementary Figure 1). Furthermore, we found that the percentage of apoptotic cells was significantly decreased by silibinin, as confirmed by the results of TUNEL staining (Fig. 3c, d), greatly increased expression of Bcl-2, and lower Bax expression (Fig. 3e–g). In summary, these results indicated cytoprotective effects of silibinin against AGE-induced osteoblasts apoptosis.

### Effects of MitoQ, CsA, and silibinin on AGE-induced mitochondrial related events

**Fig. 4:**
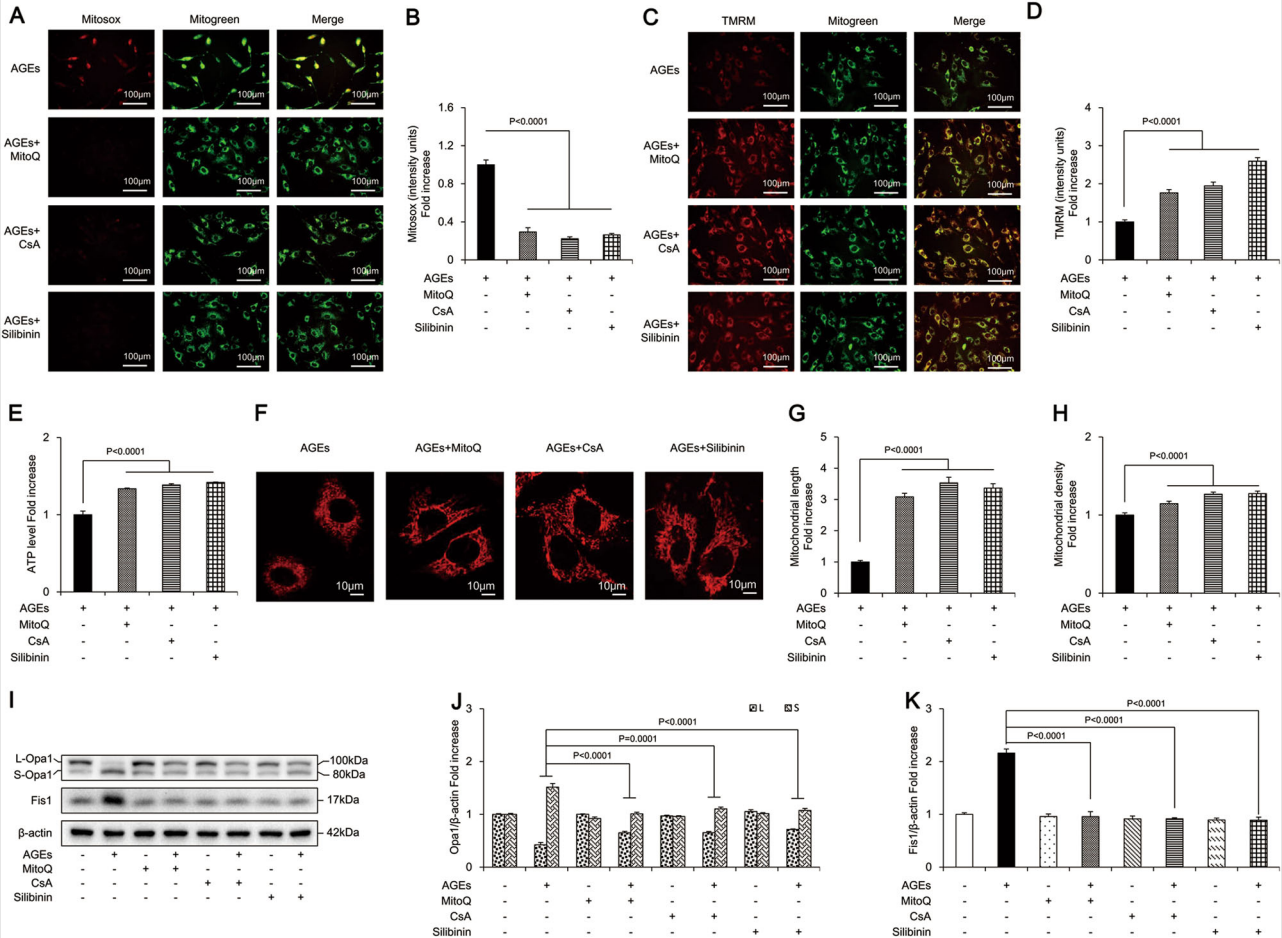
MitoQ, CsA, and silibinin attenuated mitochondrial abnormalities in osteoblastic MC3T3-E1 cells. **a**, **b** Representative images showing MitoSOX staining and quantification in the indicated groups. Scale bars = 100 μm. **c**, **d** Representative images showing TMRM staining and quantification in the indicated groups. Scale bars = 100 μm. **e** ATP production in the indicated groups. **f**–**h** Representative images of mitochondrial morphology and measurements of mitochondrial length and density in the indicated groups. Error bars indicate SEM (*n* = 15). Scale bars = 10 μm. **i**–**k** Representative immunoreactive bands for Opa1 and Fis1 in osteoblastic cells in the presence of AGEs. Quantification of immunoreactive bands for Opa1 and Fis1 relative to β-actin. Error bars indicate SEM (*n* = 6). Full-length blots are presented in Supplementary Figure 4

MitoQ significantly attenuated mtROS level (Fig. 4a, b), increased mitochondrial membrane potential (Fig. 4c, d), and restored the ATP synthesis reduced by AGEs (Fig. 4e). In addition, MitoQ protected mitochondrial morphology from the detrimental effects of AGEs (Fig. 4f, h). MitoQ also significantly increased expression of L-Opa1, and decreased the level of S-Opa1 and Fis1 (Fig. 4i–k), suggesting attenuation of mitochondrial fragmentation. These results indicated that MitoQ prevented mitochondrial abnormalities in osteoblasts subjected to mtROS.

As shown in Fig. 4a–e, CsA markedly prevented the rise in mtROS generation induced by AGEs and restored mitochondrial membrane potential and ATP production. Furthermore, CsA rescued the morphological abnormality and perturbations of mitochondrial fission/fusion proteins elicited by AGEs (Fig. 4f–k). These results indicated that mPTP opening-associated loss of mitochondrial membrane potential had a critical role in AGE-induced mitochondrial dysfunction.

Similarly, silibinin antagonized the detrimental effects of AGEs. It ameliorated mtROS generation and restored mitochondrial membrane potential and ATP production by 74%, 160%, and 42%, respectively (Fig. 4a–e). Furthermore, silibinin efficiently abolished AGE-induced morphological alterations of mitochondria, as indicated by increased mitochondrial length and density (Fig. 4f–h). Abnormal mitochondrial fission/fusion events were also ameliorated by silibinin (Fig. 4i), as revealed by markedly increased expression of L-Opa1 and reduced levels of S-Opa1 and Fis1 after its administration (Fig. 4j, k). Collectively, these results indicated that silibinin demonstrated efficient anti-oxidative and mitochondria-protective effects against AGE-induced osteoblastic cell apoptosis.

### RAGE inhibitor prevented AGE-induced apoptosis of osteoblastic MC3T3-E1 cells

**Fig. 5:**
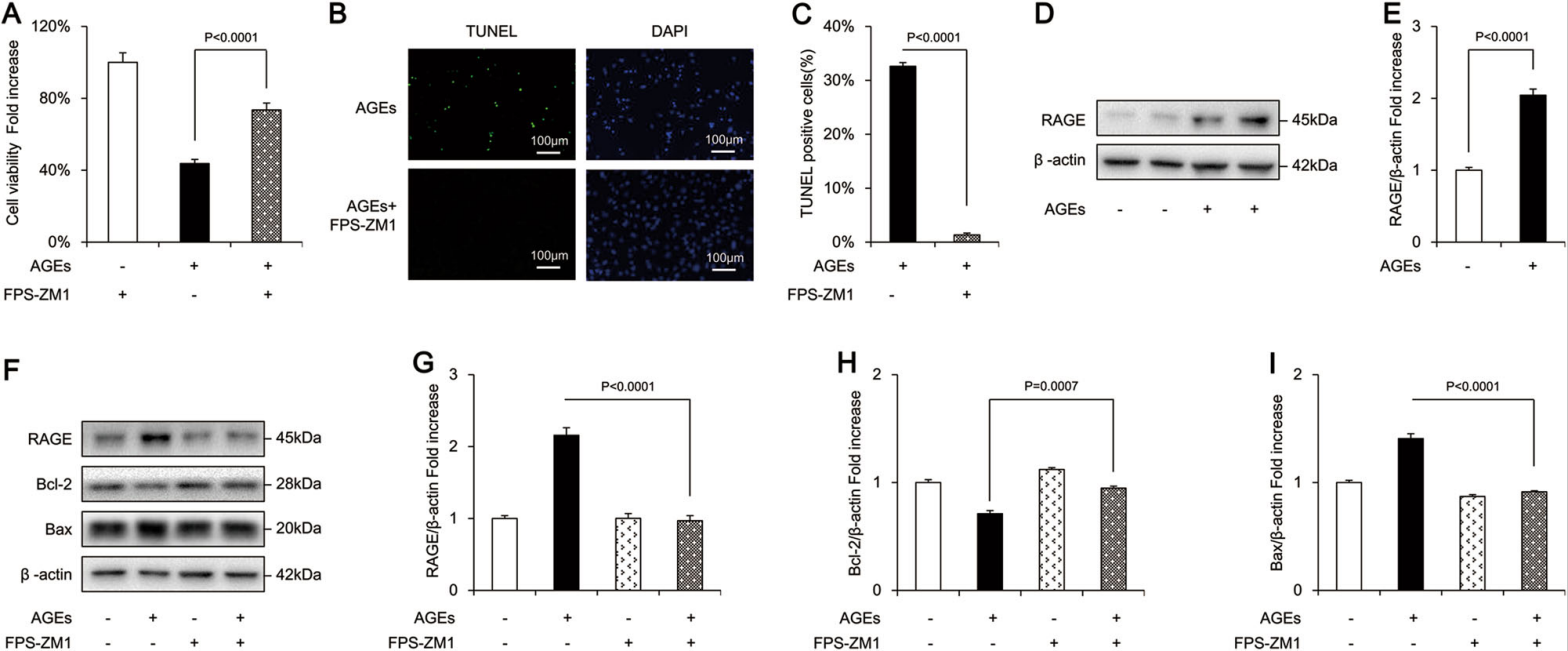
FPS-ZM1 attenuated AGEs-induced apoptosis in osteoblastic MC3T3-E1 cells. **a** Cell viability determined by MTT reduction in osteoblastic cells in the presence of AGEs with or without FPS-ZM1. Error bars indicate SEM (*n* = 6). **b**, **c** TUNEL staining and assay after FPS-ZM1 treatment. Error bars indicate SEM (*n* = 300). Scale bars = 100 μm. **d**, **f** Representative immunoreactive bands for RAGE, Bcl-2, and Bax in osteoblast with (+) or without (−) FPS-ZM1 treatment in the presence of AGES (+) or culture medium (−). Full-length blots are presented in Supplementary Figure 4. **e**, **g**, **h**, **i** Quantification of immunoreactive bands for RAGE, Bcl-2, and Bax relative to β-actin. Error bars indicate SEM (*n* = 6)

To determine whether a RAGE-mediated signaling pathway was involved in AGE-induced osteoblastic cell apoptosis, we treated cells with FPS-ZM1, a high-affinity RAGE-specific inhibitor. FPS-ZM1 markedly improved the cell viability that was reduced by AGEs (Fig. 5a). FPS-ZM1 also significantly attenuated AGE-induced cell apoptosis, as confirmed by TUNEL staining (Fig. 5b, c). In addition, FPS-ZM1 reduced the expression of RAGE, recovered Bcl-2 protein expression, and decreased the level of Bax (Fig. 5d–i).

### RAGE inhibitor attenuated AGE-induced mitochondrial abnormalities in osteoblastic MC3T3-E1 cells

**Fig. 6:**
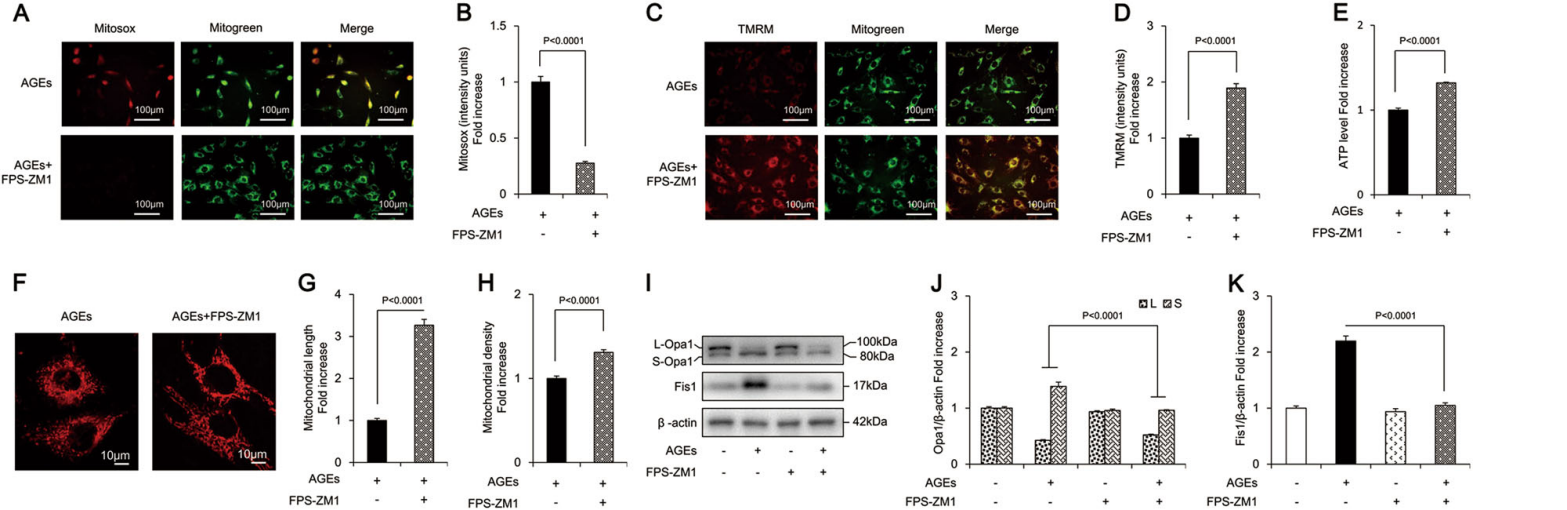
FPS-ZM1 attenuated AGEs-induced mitochondrial abnormalities in osteoblastic MC3T3-E1 cells. **a**, **b** Representative images showing MitoSOX staining and quantification in the indicated groups. Scale bars = 100 μm. **c**, **d** Representative images showing TMRM staining and quantification in the indicated groups. Scale bars = 100 μm. **e** ATP production in the indicated groups. **f**–**h** Representative images of mitochondrial morphology, and measurements of mitochondrial length and density in the indicated groups. Error bars indicate SEM (*n* = 15). Scale bars = 10 μm. **i**–**k** Representative immunoreactive bands for Opa1 and Fis1 in osteoblastic cells in the presence of AGEs. Quantification of immunoreactive bands for Opa1 and Fis1 relative to β-actin. Error bars indicate SEM (*n* = 6). Full-length blots are presented in Supplementary Figure 4

As shown in Fig. 6a–e, FPS-ZM1 significantly suppressed mtROS level, increased the mitochondrial membrane potential and ATP production. Electron microscopy further confirmed that FPS-ZM1 preserved mitochondrial morphology by maintaining mitochondrial length and shape (Fig. 6f–h). FPS-ZM1 also increased the level of L-Opa1 and reduced the expression of S-Opa1 and Fis1 (Fig. 6i–k), which indicated that mitochondrial fragmentation was attenuated. Taken together, these data demonstrated that RAGE was a major mediator of mitochondrial abnormalities involved in AGE-induced osteoblast apoptosis.

### Silibinin prevented AGE-induced apoptosis of osteoblastic MC3T3-E1 cells by directly suppressing RAGE expression

**Fig. 7:**
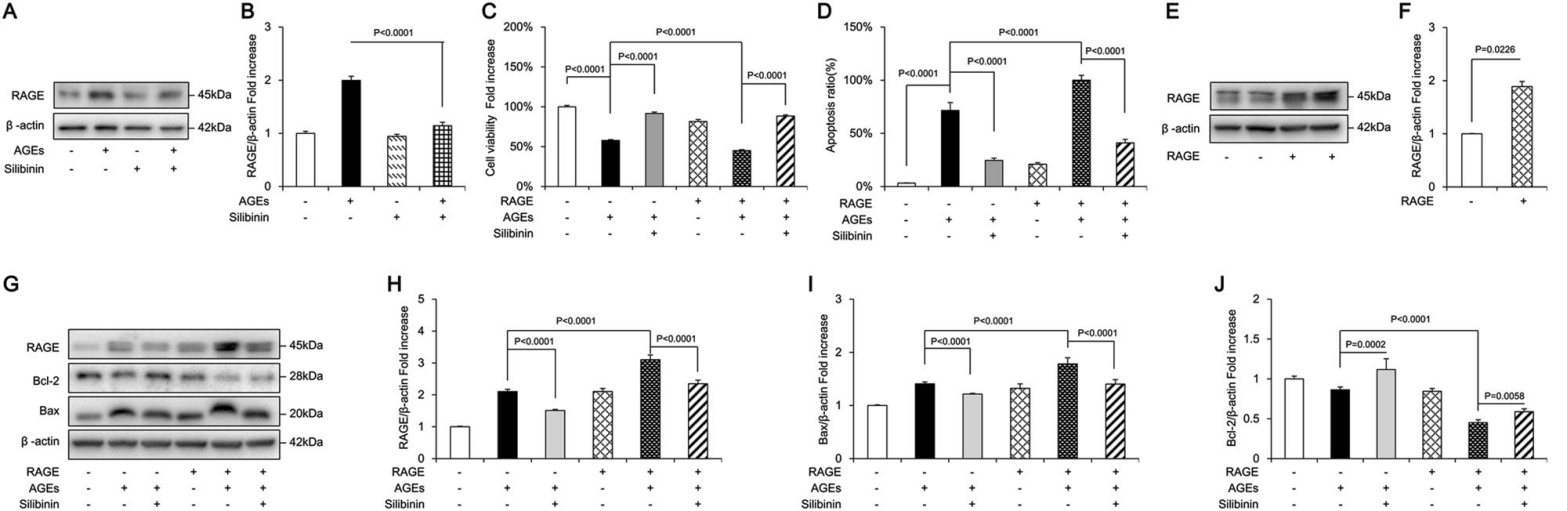
Silibinin protects AGEs-induced osteoblastic MC3T3-E1 cells apoptosis through modulation of RAGE. **a, b** Representative immunoreactive bands and relative levels of RAGE in osteoblastic cells with (+) or without (−) silibinin treatment in the presence of AGEs (+) or culture medium (−). **c** Cell viability determined by MTT reduction in osteoblastic cells treated with (+) or without (−) silibinin in the presence of AGEs (+). Error bars indicate SEM (*n* = 6). **d** Hoechst staining assay in the indicated groups. Error bars indicate SEM (*n* = 300). **e**, **f** Representative immunoreactive bands and relative levels of RAGE in osteoblastic cells transfected with plasmids carrying empty vectors or RAGE genes. **g** Representative immunoreactive bands for RAGE, Bcl-2, and Bax in in the indicated groups; **h**–**j** Quantification of immunoreactive bands for RAGE, Bcl-2, and Bax relative to β-actin. Error bars indicate SEM (*n* = 6). Full-length blots are presented in Supplementary Figure 4

Since RAGE had been shown to participate in AGE-induced osteoblast apoptosis, we next investigated whether silibinin could prevent apoptosis via modulation of RAGE. We found that silibinin directly downregulated the protein expression of RAGE (Fig. 7a, b) and greatly attenuated AGE-induced apoptosis in osteoblastic cells, as the reduced cell viability was largely rescued (Fig. 7c), and the cell apoptosis rate was significantly decreased (Fig. 7d). In addition, silibinin reduced the expression of pro-apoptotic Bax and ameliorated AGE-induced suppression of Bcl-2 protein synthesis (Fig. 7g–j).

To further explore the interference of silibinin with RAGE, we induced overexpression of RAGE in MC3T3-E1 cells using plasmid-mediated gene transfer. The cells were transfected with plasmids carrying the RAGE gene or empty vectors. Lowest cell viability and highest rate of apoptosis were detected in the group of MC3T3-E1 cells subjected to RAGE overexpression along with AGE treatment, as confirmed by the results of MTT assay (Fig. 7c) and Hoechst staining (Fig. 7d). Moreover, the RAGE transfection + AGE treatment group demonstrated significantly higher levels of RAGE and anti-apoptotic Bcl-2 but lower Bax expression than the other groups (Fig. 7e-j). These results further demonstrate that the enhanced RAGE expression mediated by RAGE overexpression was also reduced by silibinin. However, the protective effect of silibinin against AGE-induced osteoblastic cell apoptosis was partly abrogated by RAGE overexpression. These data indicated that silibinin attenuated AGE-induced apoptosis in osteoblasts through direct suppression of RAGE expression.

### Silibinin prevented AGE-induced apoptosis of osteoblastic MC3T3-E1 cells through RAGE-dependent mitochondrial pathway

**Fig. 8:**
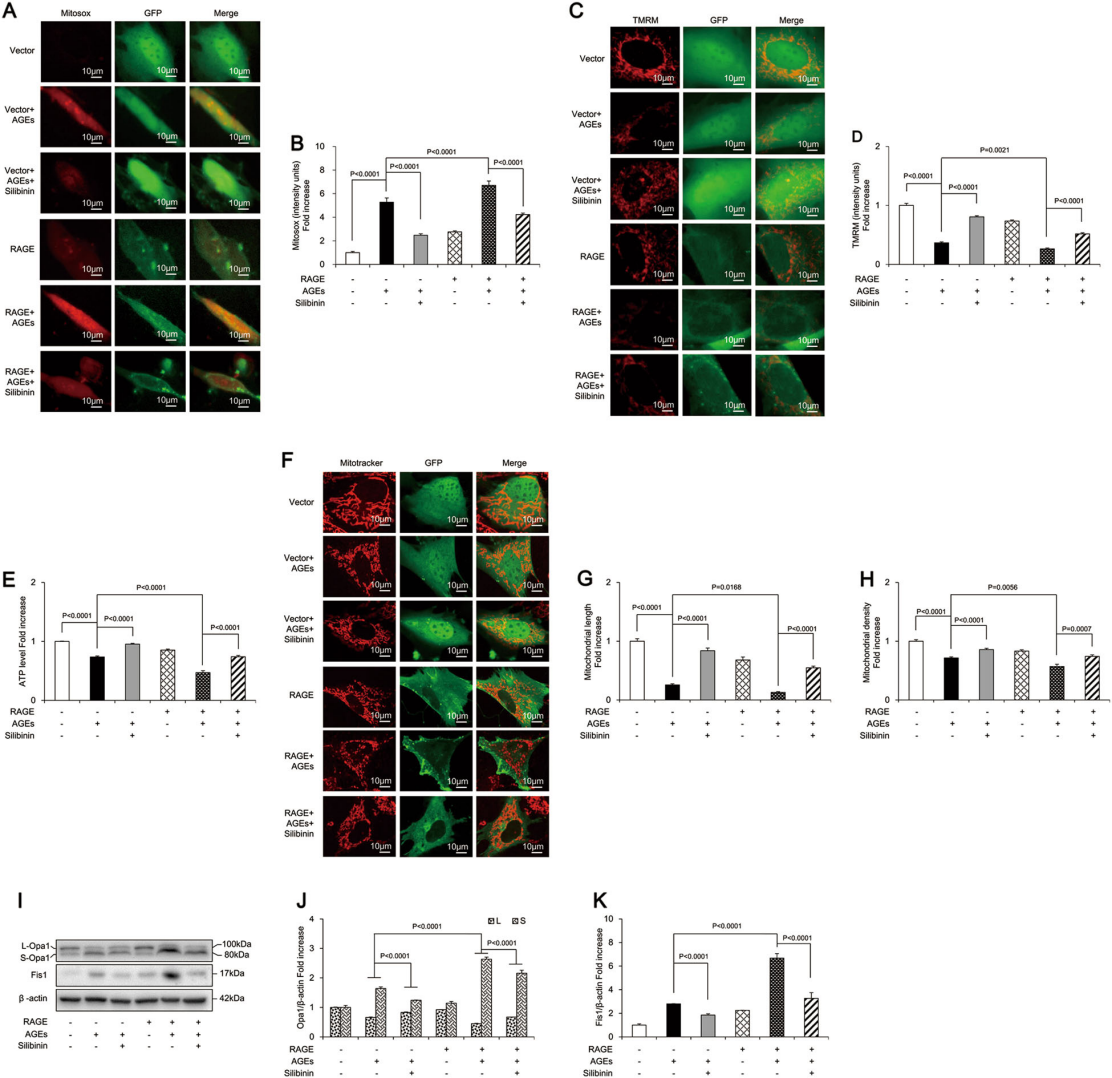
Silibinin prevented AGEs-induced osteoblastic MC3T3-E1 cells apoptosis through RAGE-dependent mitochondrial pathway. **a**, **b** Representative images showing MitoSOX staining and quantification in the indicated groups. Scale bars = 10 μm. **c**, **d** Representative images showing TMRM staining and quantification in the indicated groups. Scale bars = 10 μm. **e** ATP production in the indicated groups. **f**–**h** Representative images of mitochondrial morphology, and measurements of mitochondrial length and density in the indicated groups. Error bars indicate SEM (*n* = 15). Scale bars = 10 μm. **i** Representative immunoreactive bands for Opa1 and Fis1 in osteoblastic cells in the presence of AGEs. Full-length blots are presented in Supplementary Figure 4. **j**, **k** Quantification of immunoreactive bands for Opa1 and Fis1 relative to β-actin. Error bars indicate SEM (*n* = 6)

The above results confirmed that silibinin prevented apoptosis via direct modulation of RAGE and mitochondrial protection. We further explored whether the beneficial effects of silibinin were mediated by the regulation of a RAGE-dependent mitochondrial pathway. Our results demonstrated that RAGE transfection aggravated mitochondrial morphologic abnormality and dysfunction, as evidenced by significantly increased mtROS production (Fig. 8a, b) and reduced MMP and ATP production (Fig. 8c–e), as well as decreased mitochondrial length and density (Fig. 8f–h). RAGE transfection also altered mitochondrial dynamics, as indicated by the significant upregulation of Fis1 and S-Opa1 and downregulation of L-Opa1 (Fig. 8i–k). The present study thus provides strong evidence that silibinin prevented AGE-induced mitochondrial abnormalities in osteoblastic MC3T3-E1 cells. However, this protective effect of silibinin was largely abolished in the RAGE overexpression group (Fig. 8a–k). Taken together, these findings suggested that silibinin protected osteoblastic cells against apoptosis via a RAGE-dependent mitochondrial mechanism.

## Discussion

AGEs can directly cause apoptosis of osteogenic cells and have a critical role in the pathophysiology of diabetic osteoporosis^6,33^. Moreover, OS and OS-associated mitochondrial dysfunction are closely related to the apoptosis of osteogenic cells^15,18,19^.However, it is unclear whether OS and mitochondrial abnormalities are involved in AGE-induced osteoblast apoptosis. In the current study, we first identified a pivotal role of mitochondrial OS, dysfunction, abnormal morphology, and altered dynamics in AGE-induced apoptosis of osteoblastic cells. We also confirmed that these mitochondrial alterations were mainly mediated by RAGE. Furthermore, these pathological events can be effectively attenuated by silibinin through modulation of RAGE-dependent mitochondrial abnormalities. With these findings, the present study revealed that silibinin directly targeted RAGE and suppressed RAGE-mediated mitochondrial abnormalities, thereby preventing AGE-induced osteoblast apoptosis.

AGEs are a crucial pathogenic factor involved in diabetic complications, and their negative effects on bone tissue are well documented^4,33^. One major mechanism of these effects involves AGEs-induced excessive ROS generation that leads to osteoblast apoptosis. In the present study, decreased viability and enhanced apoptosis were also observed in AGE-treated osteoblastic MC3T3-E1 cells, which is consistent with previous studies^34^. Furthermore, AGEs significantly increased mtROS production in MC3T3-E1 cells. A previous study investigating the role of OS in AGE-treated osteoblasts only evaluated intracellular ROS production by the dihydrorhodamine 123 method^35^. Our study is the first to detect the ROS level in mitochondria by MitoSOX staining, and our findings clearly demonstrate the important role of mtROS in AGE-induced osteoblast apoptosis. Furthermore, we investigated the role of MitoQ, a widely used antioxidant designed to accumulate within mitochondria where it is rapidly converted into ubiquinol, which acts as the active antioxidant^36^. We found that MitoQ significantly attenuated AGE-induced osteoblast apoptosis, suppressed mtROS production, recovered mitochondrial morphology and dysfunction, and rescued the perturbations in mitochondrial fission/fusion proteins. These results revealed a protective effect of MitoQ against the mitochondrial abnormalities in osteoblastic cells and further confirmed the crucial role of mitochondrial OS in AGE-induced osteoblast apoptosis.

Mitochondrial dysfunction resulting from OS is commonly considered an initiating stimulus and early event in apoptosis^37^. We provided strong evidence that impaired mitochondrial function has a critical role in AGE-induced osteoblast apoptosis. Balanced mitochondrial dynamics is critical to the maintenance of functional mitochondria, energy generation, and prevention of apoptosis^17,38^. Our results indicated that AGEs-induced imbalances in mitochondrial fission and fusion in osteoblasts. Fis1 is an important mitochondrial complex that is indirectly involved in mitochondrial fission via binding of Drp1. Overexpression of Fis1 enhances mitochondrial fragmentation under a range of conditions including apoptosis^16^. In our study, an increased level of Fis1 was detected in AGE-induced osteoblastic cell apoptosis, suggesting a shift to mitochondrial fission. Blockade of Fis1, shifting the dynamic balance to favor fusion, has been proved to prevent apoptosis^39^. Therefore, downregulation of Fis1 may be a promising therapy to promote resistance to AGE-induced osteoblastic cell apoptosis. Opa1, a key mitochondrial fusion protein, is also reported to be an important participant in apoptosis. It exists in L-Opa1 and S-Opa1 forms, generated by processing at specific sites^40^. The preservation of a stable pool of L-Opa1 was needed to maintain sufficient mitochondria content and preserve cell viability^41^. In response to a pro-apoptotic stimulus, L-Opa1 is processed into S-Opa1, thereby disrupting the balance of these two forms, inducing mitochondrial fragmentation and apoptosis^42^. In the present study, we found that AGEs promoted rapid cleavage of L-Opa1 into S-Opa1 in osteoblasts, leading to loss of fusion-active L-Opa1, enhanced mitochondrial fragmentation, and subsequent apoptosis. No significant changes were found in total Drp1, Mfn1, or Mfn2 protein levels in AGE-treated osteoblasts. The functional consequences of these three proteins depend on factors including the status of modifications such as phosphorylation, type of upstream kinase enzymes, and interplay with other proteins^43,44^. Consequently, further experiments are required to elucidate the significance of activated Drp1, Mfn1, and Mfn2 in AGE-treated osteoblasts. Taken together, these results indicated that the disruption of mitochondrial dynamics was involved in the pathogenesis of AGE-induced apoptosis, which suggests that modulators of mitochondrial dynamics could have therapeutic value in the treatment of diabetic osteoporosis.

An early and key event during apoptosis is mPTP opening, which leads to the release of apoptogenic factors into the cytosol and dissipation of mitochondrial membrane potential. In our study, loss of mitochondrial membrane potential was observed in AGE-induced osteoblast apoptosis. We further pretreated the cells with CsA, which prevented the opening of mPTP by inhibiting the activity of cyclophilin D, an important component of mPTP. We found that CsA retarded the loss of mitochondrial membrane potential and rescued osteoblasts from the effects of AGEs by reversing mitochondrial damage. These findings suggest that mitochondrial membrane potential loss has an important role in the osteoblast apoptosis induced by AGEs. Thus, in future studies, investigation of mitochondrial membrane potential should provide more insights into the complex regulation of the apoptosis of osteoblasts exposed to AGEs.

RAGE, the main receptor of AGEs, is known to be involved in the apoptosis of various cell types^11,45^. However, studies regarding the role of RAGE in the apoptosis of osteoblasts are limited^10^. Previous studies have indicated that RAGE is an important player in OS and mitochondrial homeostasis^46^. Activation of RAGE directly induces ROS production, which establishes a redox crosstalk with mitochondria to amplify ROS production^47^. We detected both elevated RAGE expression and excessive mtROS generation in AGE-induced osteoblastic apoptosis. Furthermore, in our study, RAGE overexpression amplified AGE-induced apoptosis and associated mitochondrial abnormalities. The above results confirmed that RAGE-mediated mitochondrial damage had a crucial role in AGE-induced apoptosis of osteoblastic cells. Studies have confirmed that the interaction of AGEs with RAGE results in the propagation of OS-related signals and activation of various pathways that modulate mitochondrial events, including JNK, GSK-3β, MAPKs, and NF-κB^47,48^. More importantly, GSK-3β and NF-κB have been shown to have a crucial role of in the regulation of mitochondrial morphology and dynamics^49,50^. Thus, further experiments are needed to explore whether these signaling pathways are related to the RAGE-mediated dynamic disruption of mitochondria in AGE-induced osteoblastic cell apoptosis.

Silibinin exerts strong bone-protective effects^29,51^, however the effect of silibinin on osteoblastic apoptosis has not been clarified before. In the present study, we demonstrated that silibinin protected osteoblasts from AGE-induced apoptosis. Evidence has also suggested that silibinin exerts beneficial effects against mitochondrial damage under pathological conditions^24,25^. Our results revealed that the protective effects of silibinin against AGEs-induced apoptosis of osteoblastic cells were achieved via the attenuation of mitochondrial OS and dysfunction, restoration of mitochondrial morphology and aberrant mitochondrial fission/fusion. Furthermore, silibinin is well known for its antioxidant property, which is considered to be mainly responsible for its protective actions and has therapeutic value for numerous disorders^22,23^. Silibinin contributes to the antioxidant defenses in various ways, including direct-free radical scavenging, inhibition of specific ROS-producing enzymes, activation of antioxidant enzymes, etc. Indeed, direct scavenging of ROS is not likely to substantially contribute to the antioxidant protection afforded by silibinin^26^. Intriguingly, we found that silibinin directly downregulated RAGE signaling, as evidenced by reduced protein level of RAGE in silibinin-pretreated osteoblastic cells. Furthermore, we found that the anti-apoptotic and mitochondria-protective effects of silibinin were significantly abolished by RAGE overexpression. Taken together, RAGE and its dependent mitochondrial events were novel targets of silibinin against AGE-induced apoptosis in osteoblastic cells, and silibinin might be a promising potential candidate to promote bone recovery under diabetic conditions.

Although our results implicated mitochondrial abnormalities and protective effects of silibinin in AGEs-induced osteoblastic apoptosis, this study had several limitations. On one hand, more cell lines and primary cultured osteoblasts are needed to confirm the mitochondrial mechanisms underlying AGEs-induced cell apoptosis and further verify the effect of silibinin. On the other hand, further in-vivo studies should be performed to prove the protective effect of silibinin on bone loss in diabetic osteoporosis and corroborate the molecular mechanisms found in this study.

In conclusion, RAGE-mediated mitochondrial abnormalities were involved in AGEs-induced osteoblastic cell apoptosis. In addition, silibinin attenuated AGE-induced apoptosis of osteoblastic cells through the regulation of RAGE-dependent mitochondrial events (Supplementary Figure 3). This study not only provides a new insight into the underlying mitochondrial mechanisms related to AGE-induced osteoblastic cell apoptosis, but also lays a foundation for the clinical use of silibinin for the treatment of diabetic osteoporosis.

## Materials and methods

### Reagents

Cell culture medium and supplements were purchased from Life Technologies (Grand Island, NY, USA). Anti-B cell lymphoma-2 (Bcl-2), anti-Bcl-2-associated X protein (Bax), and anti-β-actin antibodies were obtained from Cell Signaling Technology (Beverly, MA, USA). Anti-Fis1 and anti-Mfn2 were from Sigma Aldrich (St. Louis, MO, USA). Anti-Mfn1 was from Abcam (Cambridge, MA, USA). Anti-Opa1 and anti-Drp1 antibodies were from BD Biosciences (San Jose, CA, USA). The chamber slides, goat anti-rabbit and goat anti-mouse secondary antibodies, and 4′, 6-diamidino-2-phenylindole (DAPI) were from Invitrogen (Carlsbad, CA, USA). MitoSOX Red, Tetramethylrhodamine methyl ester (TMRM), Mitotracker Green (MT Green), and Mitotracker Deep Red (all Molecular Probes, USA) were purchased from Life Technologies. Terminal deoxynucleotidyl transferase-mediated dUTP-biotin nick-end labeling (TUNEL) kits were from Roche (Mannheim, Germany). AGEs and bovine serum albumin (BSA) were from Merck Millipore (Billerica, MA, USA). Silibinin, 3-(4,5-dimethylthiazol-2-yl)-2,5-diphenyltetrazolium bromide (MTT), and annexin V-FITC apoptosis detection kits were from Sigma Aldrich (St. Louis, MO, USA). Mitoquinone (MitoQ) was from Focus Biomolecules (Plymouth Meeting, PA, USA). Cyclosporine A (CsA) was from Cell Signaling Technology (Beverly, MA, USA). FPS-ZM1 (4-chloro-*N*-cyclohexyl-*N*-(phenylmethyl)-benzamide) was purchased from Merck Millipore (Billerica, MA, USA). Adenosine triphosphate (ATP) assay kit, Hoechst 33342 staining kit, and lactate dehydrogenase (LDH) assay kit were from Beyotime Institute of Biotechnology (Shanghai, China). Lipofectamine 3000 transfection reagents were from Invitrogen (Carlsbad, CA, USA). Plasmids were purified with QIAGEN Plasmid Mega Kit (QIAGEN GmbH, Hilden, Germany).

### Cell culture

The murine osteoblastic MC3T3-E1 subclone 14 line used in this study was obtained from the American Type Culture Collection (Manassas, VA, USA). Cells were maintained in α-modified minimal essential medium supplemented with 10% fetal bovine serum (FBS) and antibiotics (100 IU/mL penicillin G and 100 ng/mL streptomycin) in a humidified incubator, at 37 °C, equilibrated with 5% carbon dioxide and 95% air. The culture medium was replenished twice per week.

### Cell treatment

Test compounds were prepared as stock solutions and diluted to the desired final concentrations immediately before use. Final concentrations of the compounds were as follows: AGEs (400 μg/mL), BSA (500 μg/mL), hydrogen peroxide (H_2_O_2_) (0.75 mM), silibinin (100 μM), MitoQ (1 μM), CsA (1 μM), and FPS-ZM1 (40 μM). The final concentration of dimethyl sulfoxide (DMSO) in the culture was <0.5% in all experiments. Cells were treated with or without AGEs and the indicated test compounds for various times, according to the experiment protocol.

### Amplification and purification of plasmid DNA

Plasmids containing RAGE-Enhanced Green Fluorescent Protein (EGFP) and vector-EGFP were purchased from GeneChem Co., Ltd. (Shanghai, China). The *Escherichia coli* DH-5α strain was used as competent cells for the transformation. The plasmids were amplified in Luria Bertani medium at 37 °C overnight. The endotoxin-free plasmid was purified using the QIAGEN Plasmid Mega Kit. The concentration and purity of plasmids were quantified using NanoDrop 2000 spectrophotometer and agarose gel electrophoresis, which confirmed plasmid integrity and quality.

### Plasmid transfection

Before transfection, cells were passaged and plated in 24-well plates at 70–80% confluence. Then cells were separated into two groups: Lipofectamine 3000+ RAGE-EGFP (RAGE group), and Lipofectamine 3000+ vector-EGFP (vector group). Transfection was carried out according to the Lipofectamine 3000 manufacturer’s instructions. The ratio of Lipofectamine 3000 to plasmid was 4:1 (2 μL:0.5 mg) per well. The mixture was incubated at room temperature for 15 min before transfection, then incubated with cells for 6 h, and finally the medium was replaced with minimum essential medium to stop the transfection reaction. Cells were collected 48 h after transfection for fluorescence observation, and 72 h after transfection for western blotting analysis. Successfully transfected host cells expressed Green Fluorescent Protein (GFP). When the percentage of GFP-positive cells was >90%, cells were subjected to western blotting analysis.

### Cell viability

Cell viability was determined by the MTT colorimetric assay. Osteoblastic MC3T3-E1 cells were plated in 96-well plates (1 × 10^4^ cells/well) and exposed to AGEs in the absence or presence of other test compounds. After incubation, the cells were washed twice with phosphate buffered saline (PBS) and incubated in 100 μL/well FBS-free medium supplemented with 10 μL MTT solution (5 mg/mL) at 37 °C. After 4 h, the supernatant was removed, and the resulting formazan crystals were dissolved in 150 μL DMSO for 20 min. The plates were then agitated for 15 s, and absorbance was measured at 570 nm using a microplate reader.

### Measurement of apoptosis by flow cytometry and TUNEL assay

Flow cytometry was performed to identify cell cycle and apoptotic cells. Annexin V labeled with fluorescein isothiocyanate and propidium iodide (1 μg/mL) was used to determine cell apoptosis and necrosis. After exposure to various experimental conditions, cells were trypsinized and labeled with fluorochromes at 37 °C, and then cytofluorometric analysis was performed with a fluorescence activated cell sorter scanner (Becton Dickinson, NY, USA).

In addition, TUNEL staining was carried out to identify the rate of apoptotic cells. For the assay, cells inoculated on a coverslip were fixed in 4% paraformaldehyde in PBS and permeabilized with 0.2% Triton X-100 in citrate buffer. Samples were incubated with TUNEL reaction mixture at 37 °C for 1 h, counterstained with DAPI, and observed with a fluorescence microscope (Leica TCS SPE, Germany). The percentage of apoptotic cells was estimated by counting a total of 300 cells from random fields.

### Hoechst staining

Osteoblastic MC3T3-E1 cells were treated as indicated. The cells grown on poly-l-lysine-coated glass cover slips were stained using a Hoechst 33342 staining kit. Briefly, the cells were fixed with 4% paraformaldehyde for 20 min at room temperature. Then the cells were washed two times with PBS for 3 min per wash. The medium was then aspirated and the cells were stained with Hoechst solution (1.2 μg/mL in PBS) for 5 min at room temperature in the dark. Subsequently, the cells were washed twice with PBS and immediately mounted with anti-fade fluorescence mounting medium and examined under a fluorescence microscope (Leica TCS SPE, Germany) at a magnification of ×200. Live cells exhibited dispersion and uniform fluorescence in nuclei, while dead cells were not dyed by Hoechst staining. When apoptosis occurs, marked nuclear morphological changes may be observed in the nucleus or cytoplasm, including blue fluorescent-stained compact particulates. The cells with three or more fluorescent DNA fragments were identified as apoptotic cells. Bright condensed and fragmented nuclei were calculated as the ratio of apoptotic nuclei to the total number of nuclei.

### Western blot analyses

After experimental incubations as indicated above, cells were collected and homogenized in cell lysis buffer (Cell Signaling Technology, Beverly, MA, USA). Protein concentrations were determined using a Bradford protein assay kit (Thermo Fisher Scientific, USA). Proteins were separated by a 12% sodium dodecyl sulfate-polyacrylamide gel electrophoresis and transferred to polyvinylidene difluoride (PVDF) membranes (Bio-Rad, Hercules, CA, USA). The PVDF membranes were then blocked with 5% non-fat dry milk diluted in Tris-buffered saline (pH 7.4) containing 0.05% Tween-20 (TBST) and mildly agitated on a shaker for 90 min at room temperature. Membranes were washed two times with TBST for 3 min per wash, followed by incubations with the indicated primary antibodies overnight at 4 °C as follows: anti-Drp1 (1:2000), anti-Fis1 (1:2000), anti-Mfn1 (1:1000), anti-Mfn2 (1:2000), anti-Opa1 (1:2000), anti-Bcl-2 (1:2000), anti-Bax (1:2000), and anti-β-actin (1:8000). Then the membranes were washed three times with TBST for 5 min per wash, and incubated with the anti-mouse or anti-rabbit secondary antibody (1:5000 dilution) in 5% non-fat dry milk diluted in TBST for 60 min at room temperature. Finally, the membranes were washed three times with with TBST for 10 min per wash. The protein bands were visualized using an enhanced chemiluminescence detection kit (Thermo Fisher Scientific, USA). Quantitative densitometry was performed on the identified bands using the Bio-Rad imaging system (Bio-Rad, Hercules, CA, USA) and quantified using NIH Image J software (available in the public domain).

### LDH release assay

Extracellular LDH that is released into the culture medium is a marker for cell damage. Upon completion of treatment, LDH release was measured using LDH cytotoxicity assay kit as per the manufacturer’s instructions. In brief, Aliquots of 6 × 10^4^ cells/well were transferred to a 24-well cell culture plate for an overnight incubation. After treatment, supernatants were collected and 200 lL/well was added to a black 96-well culture plate. LDH release was measured using LDH cytotoxicity assay kit as per the manufacturer’s instructions. A450 in each well was determined using an enzyme linked.

### Functional imaging assays

Osteoblastic MC3T3-E1 cells were seeded in chamber slides at a density of 10^4^ cells/well. Cells were treated with AGEs and other test compounds for 6 h. To detect mitochondrial superoxide production, MitoSOX Red, a unique fluorogenic dye for the highly selective detection of superoxide production in the mitochondria of live cells, was used. Cells were incubated with fresh culture medium containing 2.5 μM MitoSOX for 30 min. To assess mitochondrial membrane potential, cells were treated with AGEs and other test compounds for 1 h and then co-stained with MT Green (100 nM) and TMRM (100 nM) for 30 min, as in our previous study^17^.

Mitochondria were incubated in 100 nM Mitotracker Red for 30 min at 37 °C before fixation to visualize morphology. Images were captured under a confocal microscope (Leica TCS SPE). Excitation wavelengths were 543 nm for MitoSOX, TMRM, or Mitotracker Red, and 488 nm for MT Green. Post-acquisition processing was performed with MetaMorph (Molecular Devices) and NIH Image J software for the quantification and measurement of fluorescent signals corresponding to mitochondrial length and occupied area. Mitochondrial size, density, and fluorescent intensity were quantified by an investigator blinded to experimental groups. More than 100 clearly identifiable mitochondria from 10 to 15 randomly selected cells per experiment were measured in 3 independent experiments^20^.

### Detection of ATP production

For the measurement of ATP level, whole-cell extracts were lysed in lysis buffer provided in the ATP assay kit. After centrifugation at 12,000×*g* for 5 min at 4 °C, the supernatants were transferred to a new 1.5-mL tube for ATP analysis. The luminescence from a 100 μL sample was assayed in a luminometer (Molecular Devices) together with 100 μL of ATP detection buffer. A standard curve of ATP concentrations (1 nM to 1 µM) was prepared from a known amount. All experiments were carried out in triplicate. Values were reported as the mean of three independent replicates ± standard deviation (SD).

### Data analysis

Data are presented as mean ± SD. Statistical analysis was performed using Statview software (SAS Institute, Version 5.0.1). For comparisons between multiple groups, one-way ANOVA was used followed by individual post hoc Fisher tests when applicable. *p* < 0.05 was considered statistically significant.

## Electronic supplementary material

Supplementary Figure 1

Supplementary Figure 2

Supplementary Figure 3

Supplementary Figure 4

Supplemental figure legends
