# Supplementary figures and images for: RAGE-dependent mitochondria pathway: a novel target of silibinin against apoptosis of osteoblastic cells induced by advanced glycation end products
Source: Cell Death Dis. 2018 Jun 4;9(6):674. doi: 10.1038/s41419-018-0718-3 (PMC5986782; doi:10.1038/s41419-018-0718-3)

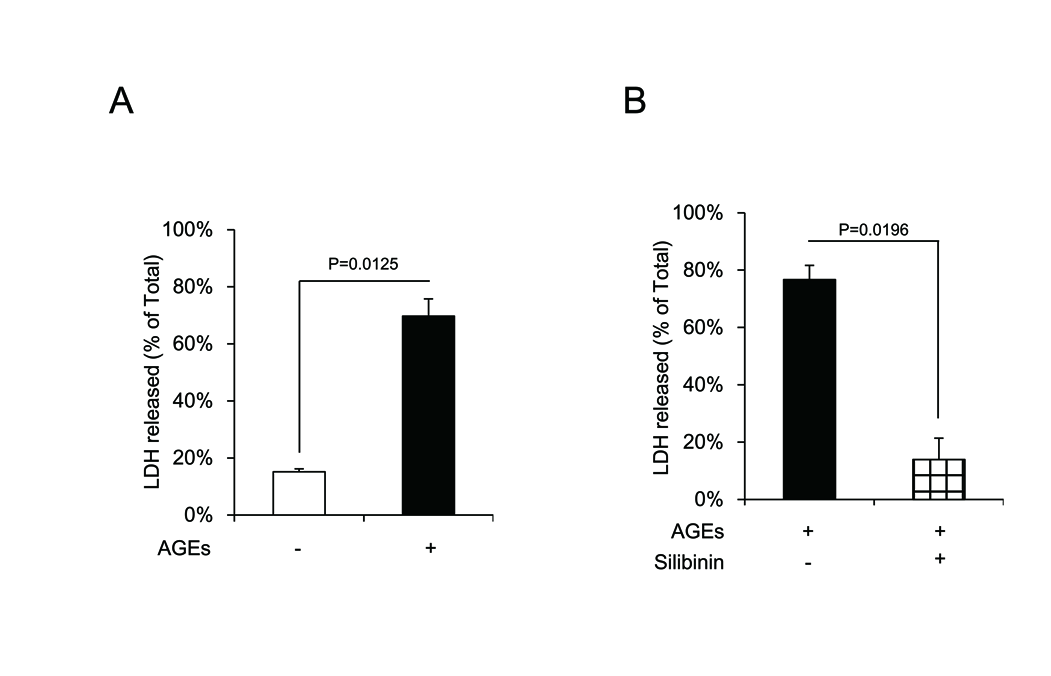

Supplement: Supplementary file 1 — Supplementary Figure 1 [file 41419_2018_718_MOESM1_ESM.tif]

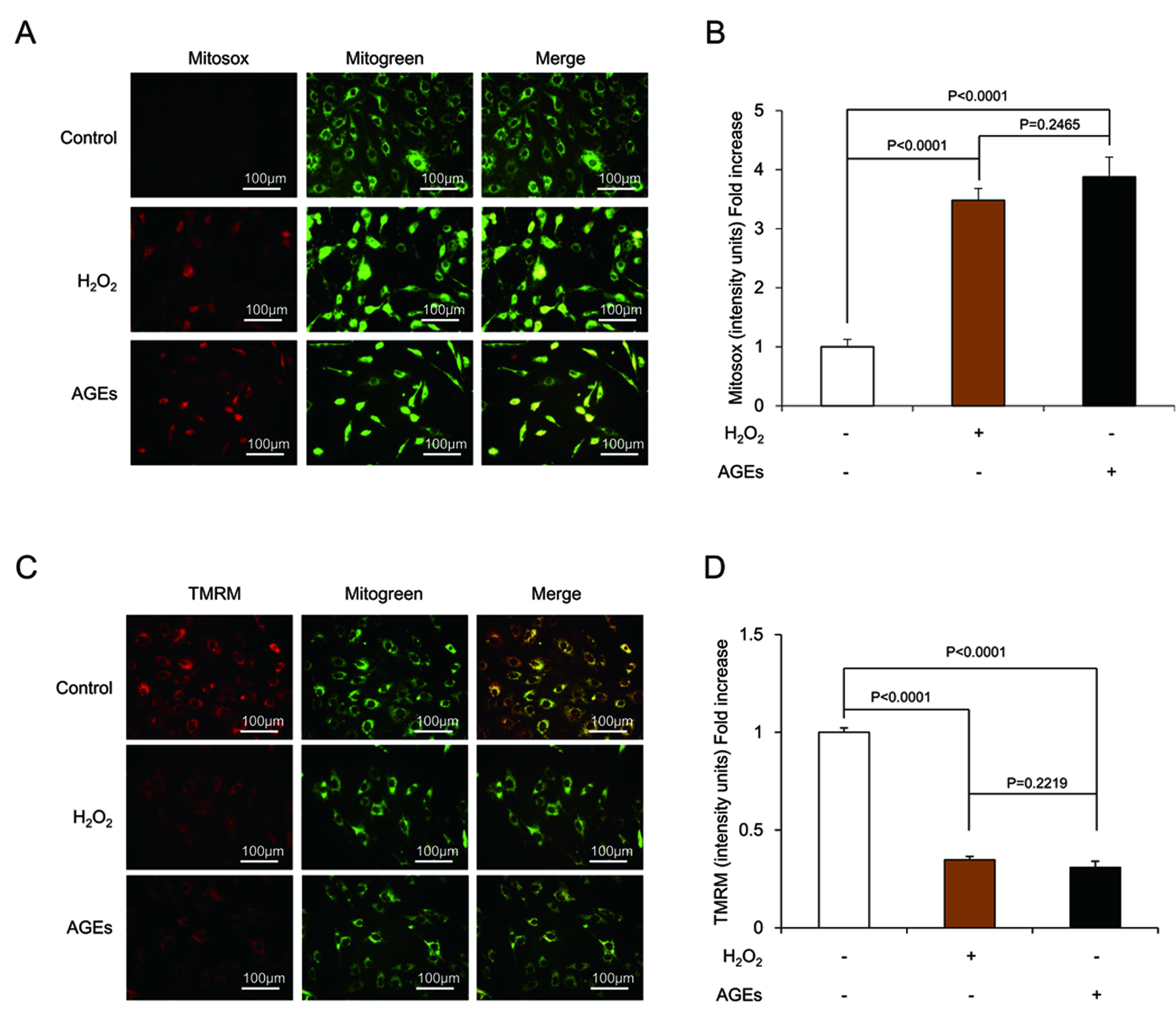

Supplement: Supplementary file 2 — Supplementary Figure 2 [file 41419_2018_718_MOESM2_ESM.tif]

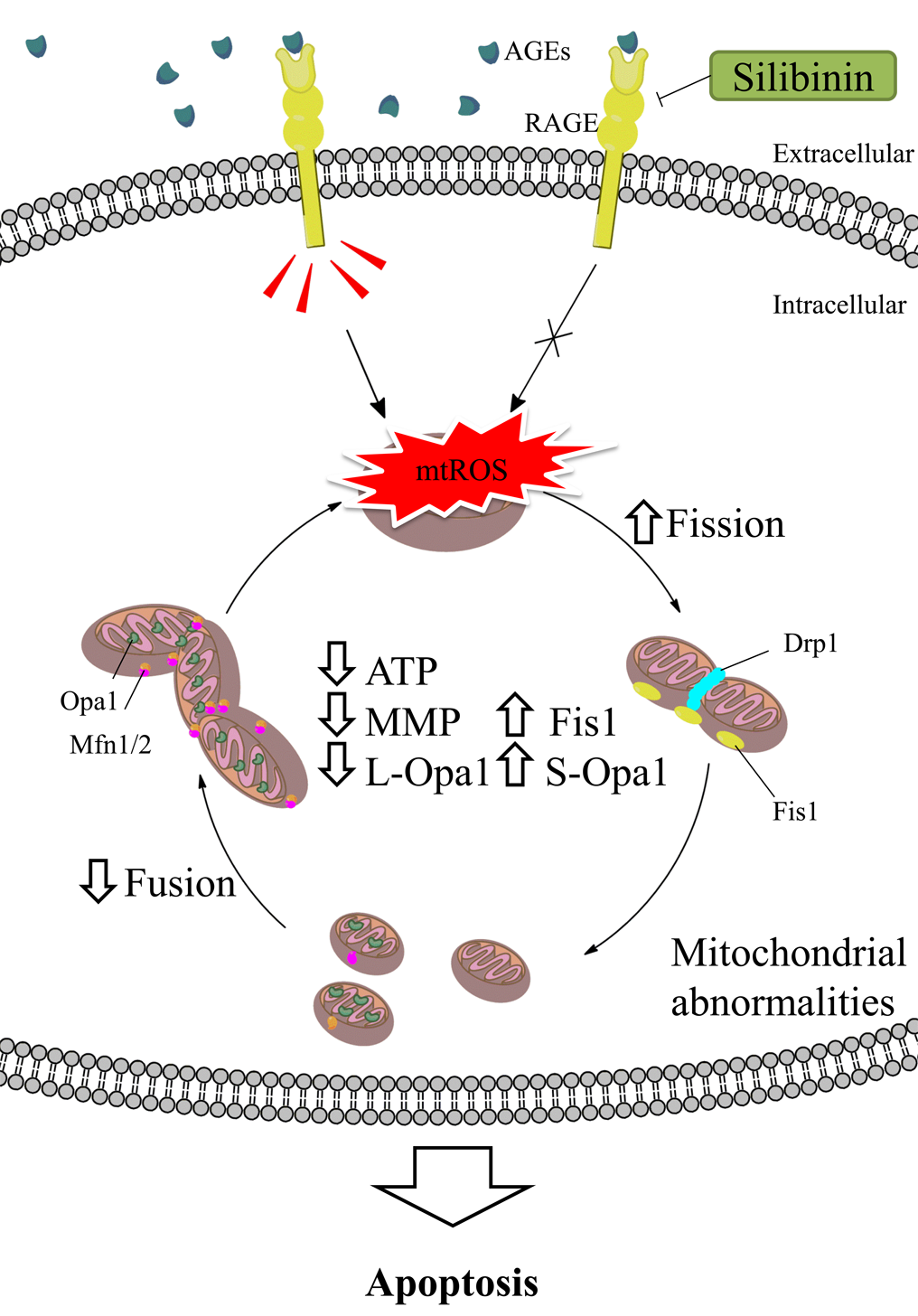

Supplement: Supplementary file 3 — Supplementary Figure 3 [file 41419_2018_718_MOESM3_ESM.tif]

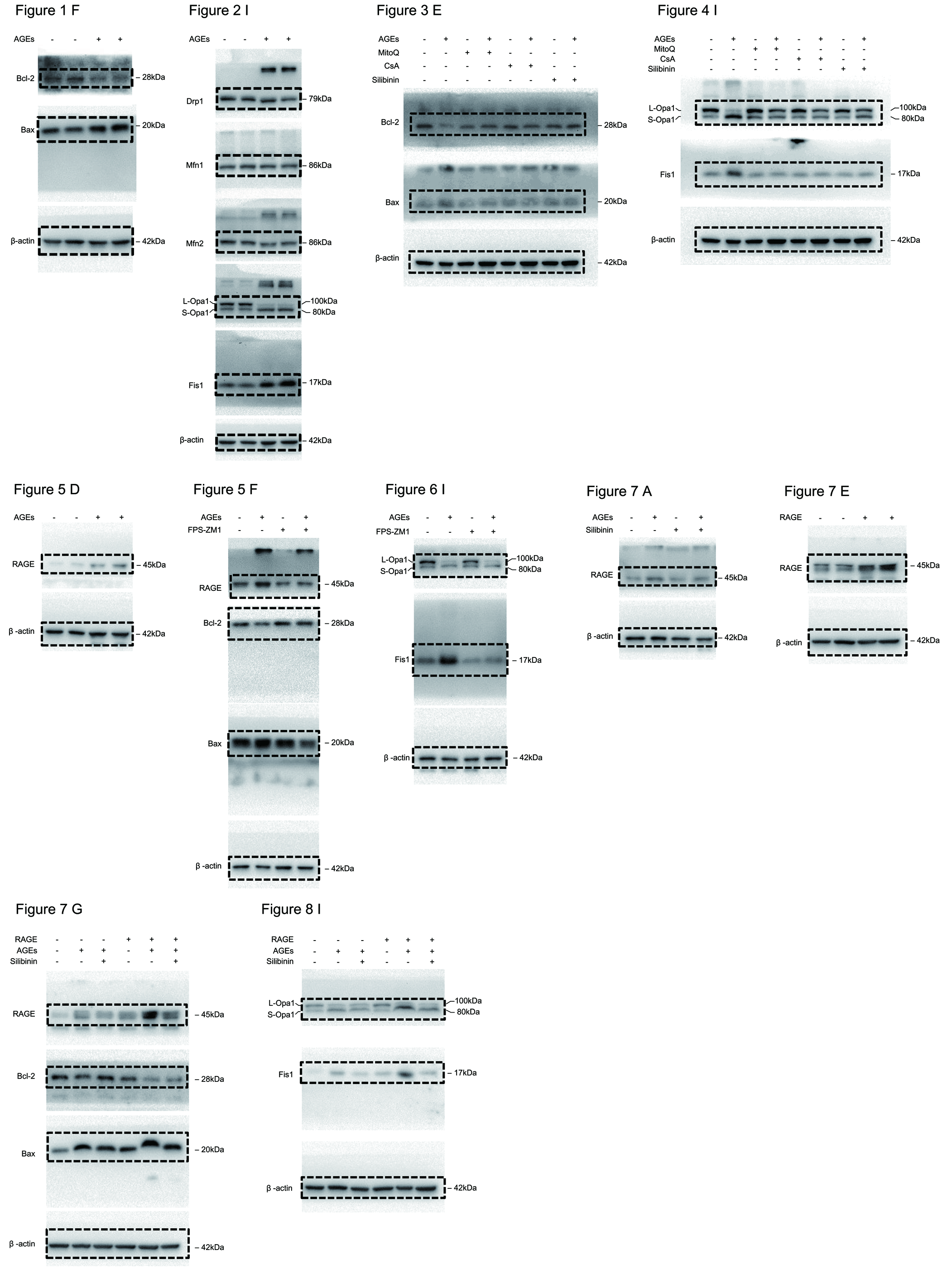

Supplement: Supplementary file 4 — Supplementary Figure 4 [file 41419_2018_718_MOESM4_ESM.tif]
