# Supplementary material for: RAGE-dependent mitochondria pathway: a novel target of silibinin against apoptosis of osteoblastic cells induced by advanced glycation end products
Source: Cell Death Dis. 2018 Jun 4;9(6):674. doi: 10.1038/s41419-018-0718-3 (PMC5986782; doi:10.1038/s41419-018-0718-3)
Supplement: Supplementary file 5 — Supplemental figure legends [file 41419_2018_718_MOESM5_ESM.docx]

**Supplementary Figure Legends**

**Supplementary Figure 1 Silibinin prevented AGEs-induced LDH release of osteoblastic MC3T3-E1 cells.** LDH released into the medium. Error bars indicate SEM (n=6).

**Supplementary Figure 2 Similar to the results of H_2_O_2_ treatment, AGEs increased the production of mitochondrial ROS and decreased mitochondrial membrane potential in osteoblastic MC3T3-E1 cells. a, b** Representative images showing MitoSOX staining and quantification in the indicated groups. Scale bars=100 μm. **c, d** Representative images showing TMRM staining and quantification in the indicated groups. Scale bars=100 μm. Error bars indicate SEM (n=6).

**Supplementary Figure 3 Working hypothesis:** Silibinin ameliorated AGEs-induced apoptosis via RAGE-dependent mitochondrial pathway.

**Supplementary Figure 4 Uncropped full-length pictures of Western blotting membranes.** Uncropped full-length pictures of Western blotting membranes presented in the main figures. Membranes were often cut to enable blotting for multiple antibodies.
